# Early recognition and safe management of unintentional intrathecal placement of an epidural catheter during combined spinal-epidural labor analgesia: a case report in the context of the 2024 OAA guidelines

**DOI:** 10.1186/s40981-026-00869-1

**Published:** 2026-07-22

**Authors:** Hidenobu Watanabe, Choko Kume, Arisa Ijuin, Yoko Yamashita, Masaki Sato, Reiko Ohara

**Affiliations:** 1 Department of Anesthesiology, National Center for Child Health and Development, 2-10-1 Okura, Setagaya-Ku, Tokyo, 157-8535 Japan; 2 Department of Anesthesiology, Kyorin University School of Medicine, 6-20-2 Shinkawa, Mitaka, Tokyo 181-8611 Japan

**Keywords:** Continuous spinal analgesia, Epidural test dose, Unintentional intrathecal catheter, Labor analgesia

## Abstract

**Background:**

Unintentional intrathecal catheter placement is a recognized complication of labor neuraxial analgesia. Although continuous spinal analgesia (CSA) may be used as a management option, prompt recognition, careful catheter management, and close monitoring are required to prevent complications such as severe spinal anesthesia.

**Case presentation:**

A 31-year-old primigravida underwent combined spinal-epidural analgesia for labor pain. Intrathecal catheter placement was diagnosed after a test dose was administered based on hypotension, motor weakness, sensory block, and glucose-positive aspirated fluid. The catheter was managed as CSA with fentanyl-based analgesia under strict monitoring. Although frequent breakthrough pain required rescue boluses, the vaginal delivery was achieved uneventfully. The patient developed a post-dural puncture headache that resolved after an epidural blood patch.

**Conclusions:**

This case illustrates the importance of early recognition of inadvertent intrathecal catheter placement and demonstrates that safe labor analgesia can be achieved through conversion to CSA with careful catheter management and close monitoring.

## Background

Unintentional intrathecal catheter placement is a rare but clinically important complication of neuraxial labor analgesia. Mistaking an intrathecal catheter for an epidural catheter can lead to the administration of excessive doses of local anesthetics, increasing the risk of high or total spinal anesthesia. Therefore, early identification of unintentional intrathecal catheter placement is essential. Management options include epidural catheter reinsertion or the use of an intrathecal catheter for continuous spinal analgesia (CSA). A recent UK survey reported that 59% of institutions chose CSA after accidental dural puncture (ADP) [1]. Furthermore, recent international guidelines and recommendations suggest that CSA may be considered an appropriate option when performed with adequate monitoring and management systems [1, 2]. Although these guidelines and surveys primarily address intrathecal catheters resulting from ADP with an epidural needle, the same principles of recognition, dosing, monitoring, labeling, and communication apply to an epidural catheter unintentionally placed intrathecally during CSEA. We apply them on that basis.

Here, we report a case of unintentional intrathecal catheter placement during CSEA, diagnosed early after a test dose and managed by conversion to CSA. We discuss its management in comparison with current international guidelines.

## Case presentation

A 31-year-old primigravida was admitted at 37 weeks and 1 day of gestation for induction of labor, because fetal bradycardia had been detected during an outpatient visit. The patient’s height was 160 cm, weight was 81 kg, and body mass index was 31.6 kg/m^2^. Her medical history was notable for severe hearing impairment; however, communication was possible when speaking clearly in a loud voice.

When cervical dilation reached 3 cm, the patient reported a Numeric Rating Scale (NRS) pain score of 7 and requested labor analgesia.

**Fig. 1:**
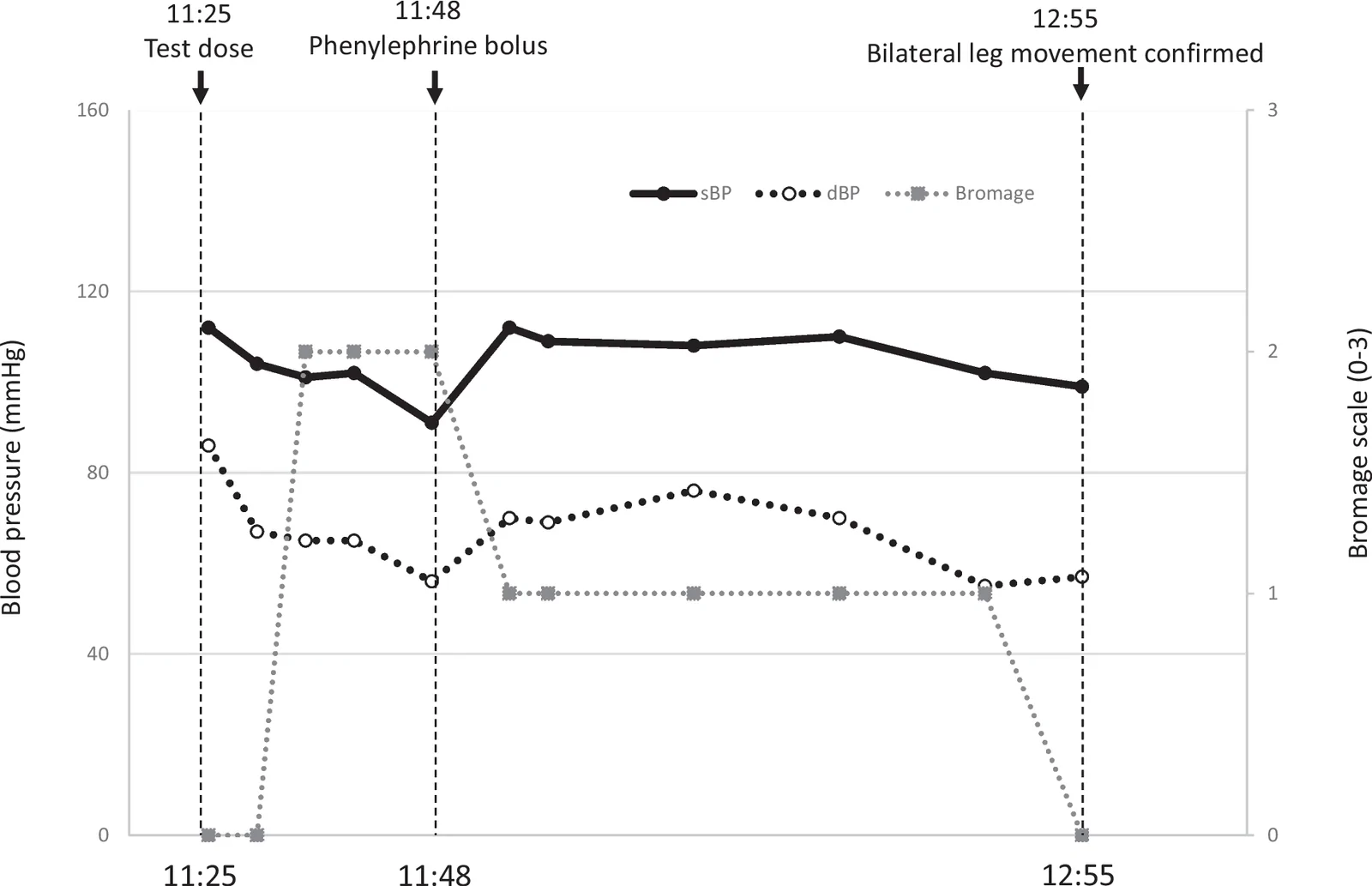
Hemodynamic status and motor block during CSEA induction. Time-course changes in blood pressure and Bromage scale acore after the administration of the test dose are shown. The patient reported an NRS pain score of 0 from the administration of the test dose until the recovery of motor function. sBP, systolic blood pressure; dBP, diastolic blood pressure; NRS, Numeric Rating Scale; Bromage scale: 0 = no motor block, 1 = unable to raise the extended leg, 2 = unable to flex the knee, 3 = unable to move the ankle

CSEA was attempted using a CSEA kit (Perifix®, B. Braun, Melsungen, Germany). The initial approach at the L3/4 interspace, with the patient in the right lateral position, was unsuccessful after three puncture attempts. A fourth attempt was made at L4/5, where the epidural space was reached via a midline approach with cephalad needle angulation. After identifying the epidural space using the loss-of-resistance technique, cerebrospinal fluid (CSF) reflux from the spinal needle was confirmed, and fentanyl 25 μg was administered intrathecally. Subsequently, after confirming the absence of CSF reflux through the epidural needle, an epidural catheter was inserted. A test dose of 3 mL of 1% lidocaine (30 mg) was administered. The epidural needle insertion depth was 7.5 cm, and the catheter was advanced 5 cm into the epidural space. Blood pressure at that time was 112/86 mmHg. Approximately 10 min after the test dose, marked analgesia and lower extremity weakness developed. Additionally, transient hypotension (91/56 mmHg) occurred and promptly improved after administration of phenylephrine 0.1 mg, with blood pressure increasing to 117/76 mmHg (Fig. 1). Concurrent clinical assessment revealed bilateral lower limb motor block (Bromage scale 2), a sensory level to cold extending to T7, and, on repeat aspiration, free flow of clear fluid from the catheter with a measured glucose concentration of 98 mg/dL, consistent with CSF. Based on these findings, the catheter was diagnosed as intrathecal.

The catheter was retained for use as an intrathecal catheter to provide CSA. The anesthesiology, obstetric, and midwifery teams were informed, verbally and in the medical record, that the catheter was intrathecal. A clearly visible label was attached to the catheter adjacent to the filter and on the infusion line.

**Fig. 2:**
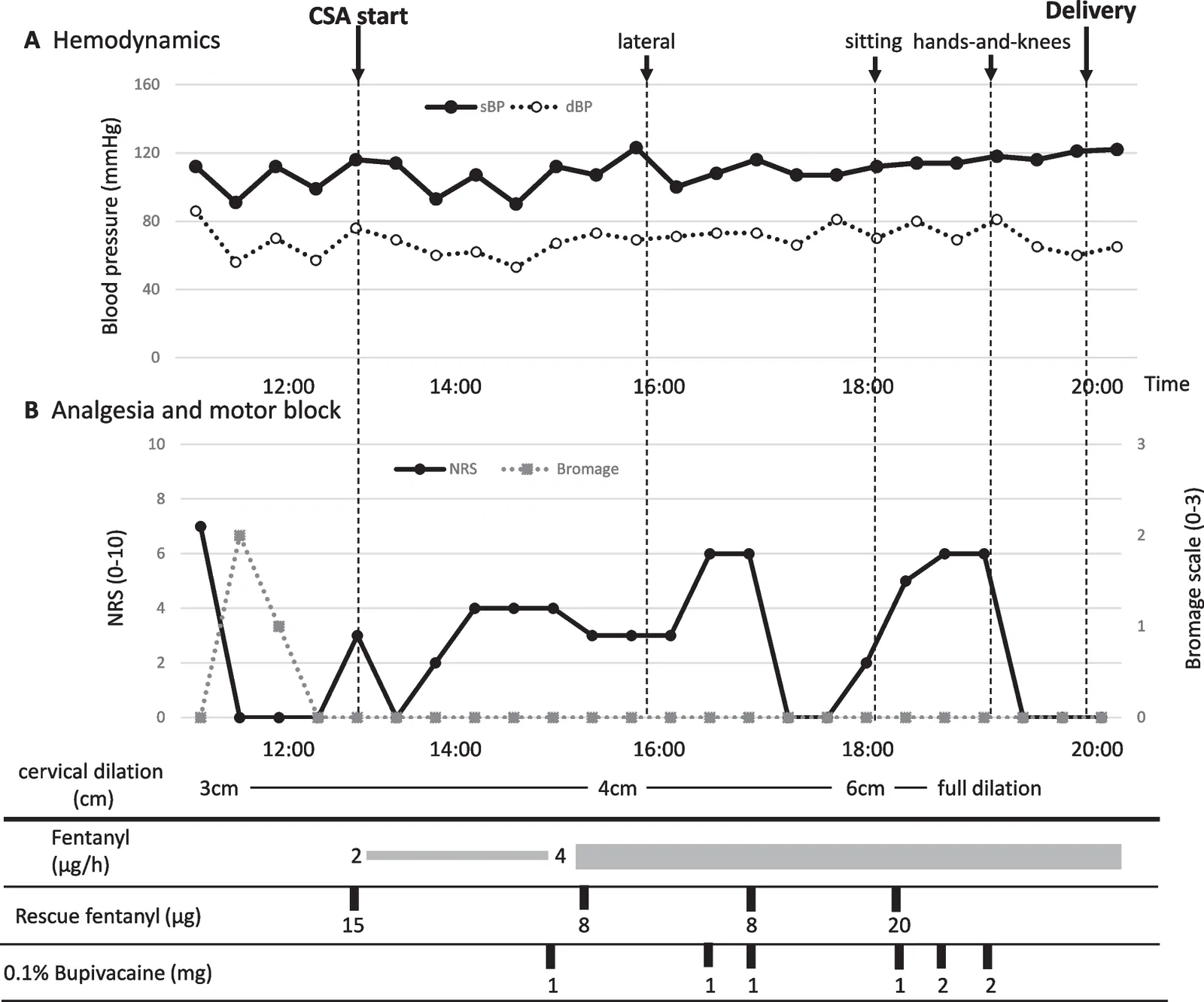
Clinical course during labor analgesia using CSA. Time-course changes in NRS, blood pressure, Bromage scale score, rescue bolus administration, and continuous fentanyl infusion rate during CSA management are shown. Rescue analgesia for breakthrough pain consisted of fentanyl 8–20 μg and/or 0.1% bupivacaine 1–2 mg. Sensory block ranged from T5 to L1 during CSA management. The lowest blood pressure recorded during the course of treatment was 91/56 mmHg at induction, with prompt recovery after phenylephrine 0.1 mg. lateral, lateral position; sitting, sitting position; hands-and-knees, hands-and-knees position; delivery, vaginal delivery; sBP, systolic blood pressure; dBP, diastolic blood pressure; NRS, Numeric Rating Scale; Bromage scale: 0 = no motor block, 1 = unable to raise the extended leg, 2 = unable to flex the knee, 3 = unable to move the ankle

After confirmation of recovery from lower extremity weakness, fentanyl 15 μg was administered through the catheter. CSA was initiated using an infusion pump (CADD-Solis®, Smiths Medical, Minneapolis, MN, USA) delivering fentanyl 2 μg/mL at 1 mL/h (2 μg/h). Maintenance with fentanyl alone was selected to avoid inadvertent administration of large doses of local anesthetics into the intrathecal space. Labor analgesia was subsequently managed with additional fentanyl and/or bupivacaine according to pain intensity (Fig. 2). Cold testing was performed hourly to assess the sensory block level. Monitoring included blood pressure every 15 min, as well as every 5 min for 15 min after each rescue bolus, with continuous pulse oximetry and cardiotocography.

No episodes of clinically significant motor block beyond Bromage 1, respiratory depression, hypotension, or signs suggestive of high spinal anesthesia were observed during CSA management (Fig. 2). The maximum sensory level to cold during CSA maintenance was T5, while the sensory block ranged between T5 and L1 throughout the course. Oxygen saturation was maintained between 97 and 99%, and the patient remained fully alert without pruritus, nausea, or vomiting. As labor progressed, the fetus developed a persistent occiput posterior position, and the patient adopted a hands-and-knees position, which she was able to do. No fetal heart rate abnormalities developed during the late stage of labor. Vaginal delivery was successfully achieved without major maternal or neonatal adverse events (Apgar scores 8 and 9 at 1 and 5 min, respectively). The neonate showed no respiratory depression and required no resuscitation. The total doses of fentanyl and bupivacaine administered were 104 μg and 8 mg, respectively. Total labor duration was 12 h and 27 min, and operative vaginal delivery was not required.

The catheter was retained until postpartum day (PPD) 2. The patient developed a post-dural puncture headache (PDPH) several hours after catheter removal. Although oral caffeine was prescribed, symptoms did not improve, and an epidural blood patch (EBP) was performed on PPD 3. After the EBP, the patient’s symptoms resolved, and she was discharged on PPD 8 with no neurological sequelae or further headache.

## Discussion

### Diagnosis of unintentional intrathecal placement of an epidural catheter: significance of the test dose

Current guidelines recommend a test dose not exceeding 10 mg bupivacaine equivalent (Table 1) [1, 2]. In the present case, 30 mg of lidocaine (approximately 7.5 mg bupivacaine equivalent [3]) was administered, which is within the recommended limit.

**Table 1:** Comparison of our intrathecal catheter management with current international recommendations

Parameter	OAA 2024 Guidelines (Griffiths et al.)	2021 Recommendations (Orbach-Zinger et al.)	This case
Test dose to confirm catheter position	Test dose ≤ 10 mg bupivacaine equivalent (Grade B)	Low-dose local anesthetic test dose	Lidocaine 30 mg via the catheter; within the recommended dose limit
Confirmation of intrathecal placement	CSF aspiration helpful but not definitive; glucose test ~ 97% accurate	Aspiration plus glucose testing; if unclear, low-dose test dose	Four findings: hypotension, Bromage 2, sensory level T7, glucose-positive aspirate (98 mg/dL)
Initial bolus for labor analgesia †	Bupivacaine up to 2.5 mg + fentanyl up to 15 μg	Bupivacaine 1.25–2.5 mg or ropivacaine 2–5 mg + fentanyl 12.5–25 μg or sufentanil 2–7 μg	Intrathecal fentanyl 25 μg only(via CSE spinal needle, before the intrathecal position of the catheter was)
Maintenance regimen †	Bupivacaine 0.1–0.125% + fentanyl 2–2.5 μg/mL as intermittent boluses (≤ 2.5 mg) or continuous infusion 1–3 mL/h	Bupivacaine 0.0417–0.1% or ropivacaine 0.1–0.2% + fentanyl 2–2.5 μg/mL or sufentanil 0.75–1 μg/mL at 1–3 mL/h	Continuous intrathecal fentanyl 2–4 μg/h only; no local anesthetic in maintenance infusion
Management of breakthrough pain †	Up to 2 mL of the maintenance solution; if ineffective, remove catheter	1–2 mL of the maintenance solution administered by trained personnel	0.1% bupivacaine 1–2 mg and/or fentanyl 8–20 μg, titrated by anesthesiologist
Maternal monitoring after first dose and boluses	NIBP every 5 min for 15 min after first dose and every bolus (Grade A); SpO₂ and ECG throughout	Frequent NIBP and continuous FHR monitoring for 30 min; then per local protocol	NIBP every 15 min during maintenance; every 5 min for 15 min after each bolus; continuous SpO₂ and CTG
Fetal heart rate monitoring	Continuous CTG throughout intrathecal analgesia (Grade B)	Continuous monitoring for 30 min after initiation, then per protocol	Continuous CTG throughout labor
Ambulation	Avoid ambulation; assist with repositioning if motor block present	Strongly discouraged during local anesthetic administration	Not ambulated; hands-and-knees position adopted in bed for fetal malposition
Catheter labelling	Clear labelling adjacent to filter and on infusion pump (Grade A)	Adequate labelling mandatory	Labeled at filter and on infusion line
Multidisciplinary communication	Verbal and written communication including at every handover (Grade A); only anesthesiologists administer top-ups (Grade A)	Communication with patient and staff; documentation in record; only anesthesiologists administer drugs	Patient, midwife, obstetric team and on-call anesthesiologist informed verbally and in medical record; only anesthetists administered drugs
Location for caesarean section top-up	Extension should be performed in the operating theatre (Grade B)	Dosing in operating theatre with full monitoring	Not applicable — vaginal delivery
Duration of catheter retention after delivery †	Remove at earliest opportunity to reduce overdose and infection risks (Grade B)	Consider leaving in situ for 24 h to reduce PDPH; precautions against drug error and CSF leak	Retained until postpartum day 2 (~ 48 h) in an attempt to reduce the risk of PDPH
Intrathecal saline injection for the prevention of PDPH	Not specifically recommended; evidence insufficient	10 mL intrathecal saline may be injected; evidence weak	Not administered
Outcome	—	—	Vaginal delivery (Apgar 8/9); no maternal cardiorespiratory complications; PDPH on PPD 2; resolved after EBP on PPD 3

*OAA* Obstetric Anaesthetists' Association, *CSE* Combined spinal-epidural, *CSF* Cerebrospinal fluid, *CTG* Cardiotocography, *EBP* Epidural blood patch, *FHR* Fetal heart rate, *NIBP* Non-invasive blood pressure, *PDPH* Post-dural puncture headache, *PPD* Postpartum day, *SpO₂* Peripheral oxygen saturation

^**†**^indicates parameters where our management diverged from current guideline recommendations

Although CSF glucose testing has an accuracy of approximately 97% [4], current guidelines emphasize that the absence of CSF aspiration does not exclude intrathecal catheter placement [1]. In this case, sufficient observation after the test dose allowed for the assessment of multiple clinical signs (hypotension, motor block, high sensory block, and glucose-positive aspirated fluid), facilitating early recognition of the intrathecal catheter placement.

### CSA regimen: comparison between current guidelines and the present case

Current guidelines recommend maintaining CSA with continuous administration of a low-concentration local anesthetic in combination with an opioid (Table 1) [1, 2]. In the present case, however, fentanyl-only maintenance was initially selected to avoid inadvertent intrathecal administration of large doses of local anesthetics. Although no major cardiorespiratory complications occurred, additional fentanyl and low-dose bupivacaine were repeatedly required as labor progressed. A similar observation was reported by Hyuga et al., in which opioid-based CSA alone did not provide sufficiently stable analgesia throughout labor, and supplemental local anesthetic administration was required [5]. This observation is consistent with these guideline recommendations and with the report by Hyuga et al., suggesting that fentanyl-only maintenance may have limited analgesic efficacy. It should be interpreted with caution, however, as it derives from a single case. The principal contribution of the present case lies not in the analgesic regimen but in the early, multimodal recognition of unintentional intrathecal catheter placement during CSEA—based on the combined assessment of motor block, sensory level, hemodynamic change, and glucose-positive aspirate after a test dose—and the subsequent conversion to CSA with guideline-concordant safety measures under strict monitoring.

In the present case, anesthesiologist-managed dosing and continuous monitoring enabled safe labor management without evidence of high spinal anesthesia.

### Prevention of PDPH and duration of catheter retention

Griffiths et al. recommend removal of intrathecal catheters at the earliest opportunity due to the risks of infection and inadvertent drug administration [1]. In contrast, Orbach-Zinger et al. suggest considering catheter retention for 24 h to reduce the risk of PDPH [2] (Table 1). In the present case, because 24 h had not yet elapsed by PPD1, the catheter was retained until PPD 2 in an attempt to prevent PDPH; however, PDPH still developed, and an EBP was ultimately required. A recent international consensus likewise concluded that intrathecal catheter placement does not clearly reduce the incidence of PDPH or the need for EBPs [6]. Decisions regarding catheter retention should therefore balance the risks of infection and medication errors.

### Importance of multidisciplinary communication

Recent guidelines repeatedly emphasize the importance of clear labeling, communication during handovers, and avoidance of drug administration by non-anesthesiologists (Table 1) [1, 2]. In the present case, these measures of multidisciplinary information sharing, documentation, and labeling helped prevent inadvertent local anesthetic administration.

In conclusion, this case highlights how early recognition of unintentional intrathecal catheter placement, clear identification of the catheter, and strict management protocols can prevent medication errors and serious neuraxial complications. Furthermore, careful management of CSA enabled safe labor analgesia and delivery.

## Data Availability

All data generated or analyzed during this study are included in this published article.

## Abbreviations

ADP: Accidental dural puncture
CSA: Continuous spinal analgesia
CSEA: Combined spinal-epidural analgesia
CSF: Cerebrospinal fluid
EBP: Epidural blood patch
NRS: Numeric Rating Scale
OAA: Obstetric Anesthetists’ Association
PDPH: Post-dural puncture headache
